# Georgia O’Keeffe (1887–1986). Cow’s Skull with Calico Roses (1932)

**DOI:** 10.3201/eid1003.AC1003

**Published:** 2004-03

**Authors:** Polyxeni Potter

**Affiliations:** * Centers for Disease Control and Prevention, Atlanta, Georgia, USA

**Figure:**
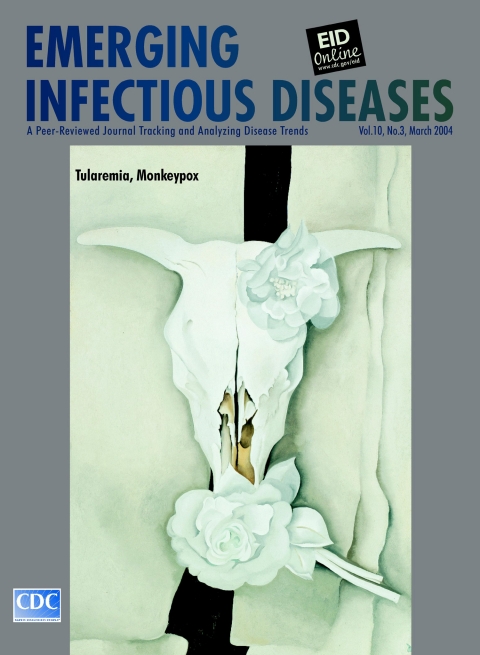
Georgia O’Keeffe (1887–1986). Cow’s Skull with Calico Roses (1932). Oil on canvas (91 cm x 61 cm). The Art Institute of Chicago

“To me they are as beautiful as anything I know,” Georgia O’Keeffe said of the sun-bleached bones and skulls she found in the desert. “To me they are strangely more living than the animals walking around….The bones seem to cut sharply to the center of something that is keenly alive on the desert even tho’ it is vast and empty and untouchable—and knows no kindness with all its beauty” (*1*). The ragged mountain terrain with its fossilized formations, saturated color, and naked wilderness held inexhaustible fascination for O’Keeffe and was a source of inspiration for most of her artistic career.

Born in Sun Prairie, Wisconsin, the second of seven children, Georgia O’Keeffe was a pioneering and charismatic woman. Training to be an art teacher, she attended the Art Institute of Chicago, Art Students League in New York, University of Virginia in Charlottesville, and Columbia University’s Teachers College. During her teaching years in Texas and later as an artist in New York City, O’Keeffe showed herself to be a complex and contradictory person with exceptional observation skills. She could fathom and depict in her work the immensity of creation in a homespun style reminiscent of what Willa Cather once called “that irregular and intimate quality of things made entirely by the human hand” (*2*).

Over the years, O’Keeffe became an “antiauthoritarian revolutionary,” the notoriety of her lifestyle sometimes overcoming the originality of her work. She shunned European traditions and influence and resisted all manner of paternalism. Like Piet Mondrian and Kasimir Malevich, she never signed her paintings, and like Jackson Pollock, she found Native American art as inspiring as Renaissance art (*3*). Eventually, she abandoned the New York City art scene that founded her reputation and moved west to New Mexico for a more authentic artistic experience. There, in a landscape unencumbered by undue neighborliness and excessive vegetation, she created work that was timeless, universal, and impersonal.

From the grandeur and vastness of the western landscape, O’Keeffe extracted a compressed, concise, and reductive style. Breaking away from the constraints of scale, she painted telescopic images that favored the distant and the immediate. She made the small seem large and the large small as she focused on a single isolated object: a mountain, a stone, a flower, a bone. Educated in oriental scroll painting and influenced by the work of Wassily Kandinsky, she understood that emptiness could signify fullness, and she applied that principle in panoramic landscape paintings, as well as in lone objects placed in pictorial space (*3*).

Like Frida Kahlo, with whom she maintained lively correspondence, O’Keeffe became intimately familiar with her subjects, wanting to merge and become one with them at the moment of creation. “I find that I have painted my life,” she confided, “…things happening in my life—without knowing” (*4*). Close acquaintance with the subject guided the accurate presentation not only of the outward image but also of the sensation within, transforming the subjective and personal to the mystical and universal.

“I have picked flowers where I found them,” O’Keeffe acknowledged, “…have picked up sea shells and rocks and pieces of wood where there were sea shells and rocks and pieces of wood that I liked….When I found the beautiful white bones on the desert I picked them up and took them home too….I have used these things to say what is to me the wideness and wonder of the world as I live in it” (*5*). Stripping things of extraneous detail, the artist reached for their essential geometry and substance and created images that were at once realistic and abstract.

The desert, the prairie, wide open spaces, O’Keeffe’s chosen world, which inspired Cow’s Skull with Calico Roses (on this month’s cover of Emerging Infectious Diseases), contained all the elements essential to her art: eternal beauty, spirituality, and a timeless connection with the past. The fragile bovine skull, a ghostly remnant, hangs stark against the funereal strip in the center of the canvas. Exquisitely fine, it shares the lyricism of the floral accents and surrounding fabric folds. Its seemingly vacant visage sends an unmistakably symbolic message of death and rejuvenation.

The mythical world of the American West has had enduring allure, and not only for its artistic potential. Its vast expanses of apparently arid land, often hermetic on the surface but teaming with life, have long fascinated the naturalist, for their dust contains eons of artifacts and clues to many of humanity’s puzzles.

Throughout most of western North America, from Canada to Mexico, infectious diseases peculiar to the region have been part of the landscape. With coccidioidomycosis, hantavirus pulmonary syndrome, and plague, among other vector-borne infections, the desert takes its toll. More recently, the region’s famed underground “communities” of myriad prairie dogs have surfaced in the news. Exposed to a viral zoonotic agent imported from across the seas and transported around the United States, prairie dogs from O’Keeffe’s adopted Southwest brought new notoriety to her native Wisconsin—site of the first outbreak of monkeypox outside the rain forests of central and western Africa.

Prairie dogs, the most social members of the squirrel family, have made their settlements from Montana to Texas and in higher elevations of the Mojave, Great Basin, and Chihuahuan deserts, posing little risk to humans as long as nature and its endemic zoonoses were in balance. Yet, import of exotic rodent species and relocation of indigenous wildlife to other areas as pets have compromised the integrity of natural cycles, proving perilous to both animal and human communities and raising the specter of interspecies transmission of infectious agents, among them those that cause tularemia and monkeypox.
